# Biodegradation of poly(l-lactic acid) and poly(ε-caprolactone) patches by human amniotic fluid in an in-vitro simulated fetal environment

**DOI:** 10.1038/s41598-022-07681-8

**Published:** 2022-03-10

**Authors:** Rigwed R. Tatu, Marc Oria, Marepalli B. Rao, Jose L. Peiro, Chia-Ying Lin

**Affiliations:** 1 Department of Biomedical Engineering, University of Cincinnati, Cincinnati, OH USA; 2 Center for Fetal and Placental Research, Cincinnati Children’s Hospital Medical Center (CCHMC), Cincinnati, OH USA; 3 Division of Pediatric General and Thoracic Surgery, Cincinnati Children’s Hospital Medical Center (CCHMC), Cincinnati, OH USA; 4 Department of Surgery, College of Medicine, University of Cincinnati, Cincinnati, OH USA; 5 Department of Environmental Health, University of Cincinnati, Cincinnati, OH USA; 6 Department of Biomedical Engineering, University of Cincinnati, Cincinnati, OH USA; 7 Department of Orthopedic Surgery, University of Cincinnati, Cincinnati, OH USA; 8 Department of Neurosurgery, University of Cincinnati, Cincinnati, OH USA

**Keywords:** Implants, Biomaterials

## Abstract

Open spina bifida or myelomeningocele (MMC) is a devastating neurologic congenital defect characterized by primary failure of neural tube closure of the spinal column during the embryologic period. Cerebrospinal fluid leak caused by the MMC spinal defect in the developing fetus can result in a constellation of encephalic anomalies that include hindbrain herniation and hydrocephalus. The exposure of extruded spinal cord to amniotic fluid also poses a significant risk for inducing partial or complete paralysis of the body parts beneath the spinal aperture by progressive spinal cord damage in-utero. A randomized trial demonstrated that prenatal repair by fetal surgery, sometimes using patches, to cover the exposed spinal cord with a watertight barrier is effective in reducing the postnatal neurologic morbidity as evidenced by decreased incidence and severity of postnatal hydrocephalus and the reduced need for ventricular-peritoneal shunting. Currently, the use of inert or collagen-based patches are associated with high costs and inadequate structural properties. Specifically, the inert patches do not degrade after implantation, causing the need for a post-natal removal surgery associated with trauma for the newborn. Our present study is aimed towards in-vitro degradation studies of a newly designed patch, which potentially can serve as a superior alternative to existing patches for MMC repair. This novel patch was fabricated by blending poly(l-lactic acid) and poly(ε-caprolactone). The 16-week degradation study in amniotic fluid was focused on tracking changes in crystallinity and mechanical properties. An additional set of designed patches was exposed to phosphate-buffered saline (PBS), as a time-paired control. Crystallinity studies indicate the progress of hydrolytic degradation of the patch in both media, with a preference to bulk erosion in phosphate buffered saline and surface erosion in amniotic fluid. Mechanical testing results establish that patch integrity is not compromised up to 16 weeks of exposure either to body fluids analog (PBS) or to amniotic fluid.

## Introduction

Open spina bifida or myelomeningocele (MMC) is a devastating neurologic congenital defect characterized by primary failure of primitive neural tube closure of the spinal column during the embryologic period^1–3^. Cerebrospinal fluid (CSF) leak caused by MMC spinal defect in the developing fetus can result in a constellation of encephalic anomalies that include skull deformity, hindbrain herniation, Chiari malformation, brainstem abnormalities, and obstructive hydrocephalus^1–3^. The exposure of the extruded spinal cord to amniotic fluid through the defect also poses a significant risk for inducing partial or complete paralysis of the body parts beneath the spinal aperture by progressive in-utero neuro-inflammation and neuronal loss of the spinal cord tissue^1–3^. A recent randomized trial demonstrated that prenatal MMC repair by fetal surgery is effective in reducing the postnatal neurologic morbidity, as evidenced by preserving motor function, reverting the hindbrain herniation, decreased incidence and severity of postnatal hydrocephalus, and reduced need for postnatal ventricular-peritoneal shunting^4^. However, as open fetal surgery has been noticed to be associated with potential for maternal–fetal morbidities, innovative fetoscopic interventions to repair MMC, first in animal models and now clinically, are receiving growing attention for their minimally invasive nature^4–7^. Nonetheless, deploying patches through small trocar ports and unfolding patches for coverage of the spinal defect can be burdensome and thus uncontrollably prolong the surgical duration and complicate the efficacy of the watertight repair.

Incumbent patches adopted in fetoscopic MMC repair are primarily commercial surgical patches. These patches are typically naturally derived (e.g. dermal, pericardium, collagen, or bio-cellulose based) that can be absorbed over time^8^. The main issues that have been identified with the biological patches are their poor mechanical properties to sustain before the wound healing process is complete^8,9^. Moreover, there have been studies revealing the risk of enzyme-activated degradation that further deteriorates the mechanical strength of such patches after in-vivo deployment^10,11^. In addition, there is a high cost associated with the fabrication of tissue-derived products^8,9,12^. Introducing synthetic patches made from silicone or Teflon seems to help address the issue for inadequate mechanical integrity, but the inert materials used pose a different set of problems. The synthetic patches are usually non-degradable, which many times lead to chronic inflammation or even infection that requires a secondary procedure for retrievals^13^. The additional procedure inevitably causes economic and psychological burdens for the patients.

This scenario makes it necessary to develop a biodegradable patch fit for prenatal or postnatal MMC repair that can retain mechanical integrity up to 16 weeks of implantation, and eventually degrade inside the body in the long term. Our group fabricated blend films of poly(lactic acid) and poly(ε-caprolactone) to fulfill these requirements and for potential use as an MMC repair patch^14,15^. Subjecting the patch to conditions identical to those encountered on implantation, provides a heightened understanding of the degradation mechanism and its effect on material properties.

The current study specifically focuses on the in-vitro degradation of the designed patch in a simulated fetal environment by using body fluid analog (PBS) and human amniotic fluid.

## Materials and methods

### Human amniotic fluid samples

Deidentified Amniotic Fluid (AF) samples from different patients were collected during fetoscopic surgeries in monochorionic twins affected by twin-to-twin transfusion syndrome (20 and 26 weeks of gestation) at the time of amnioreduction (discarded AF). These surgeries were conducted at the Cincinnati Fetal Care Center within the Cincinnati Children Hospital Medical Center (CCHMC). The experimental protocol was shared and informed consent was obtained from all subjects and/or their legal guardian(s). All the methods were performed under guidelines and regulations approved by the Ethics Committee of the Cincinnati Children’s Hospital Medical Center (CCHMC) (IRB#2017-2414).

### Patches

Patches were composed of blend films of poly(l-lactic acid) (PLA) and poly(ε-caprolactone) (PCL), fabricated by solvent casting. The blend consisted of 83% PLA and 17% PCL. The rationale behind the PLA:PCL ratio for the blend films is explained in detail in previous work^14^.

### Degradation studies

Biodegradability of the designed patch was investigated in compliance with ASTM F1635, standard test method for in-vitro degradation testing of hydrolytically degradable polymer resins and fabricated forms of surgical implants. Post-implantation, fetoscopy patches interact with amniotic fluid that protects the fetus in a pregnant uterus. Water in amniotic fluid originates from maternal plasma and advances through fetal membranes depending on hydrostatic and osmotic forces^16^. However, prior work on degradation behavior of PLA-PCL blends is restricted to phosphate buffered saline^17,18^.

We pioneered a study where 3 cm × 0.8 cm patch strips were immersed in 24 tubes filled with fresh amniotic fluid, which was replenished weekly. Amniotic fluid was extracted between 20–26 weeks of gestation from pregnant mothers and was used to replenish the tubes. The test bed of 24 tubes was subjected to multiaxial movements to simulate fetal movements in the womb, and simultaneously placed in a convection oven at 37 °C. This is the first exploration of polymer degradation in human amniotic fluid (Supplementary Fig. 1).

Property changes in amniotic fluid were tracked at time points of 4, 8, 12 and 16 weeks, as the patch is implanted 16 weeks prior to birth. As a time-paired control, 24 tubes containing patch strips were immersed in phosphate buffered saline (PBS) (pH = 7.4) and subjected to the same conditions described above. Phosphate-buffered saline also simulates physiological fluids encountered by the patch on implantation, making it the perfect addition to our degradation study. Sections of the test bed with tubes containing amniotic fluid and phosphate buffered saline were referred to as AF and PBS modules respectively. This study focused on the effect of AF and PBS exposure on the crystallinity and mechanical properties of the designed patches. An additional set of 6 patch strips was included in both AF and PBS exposure modules as a factor of safety.

#### X-ray diffraction

X-ray diffraction was used to study changes in crystallinity of the patch, i.e., PLA-PCL blend. An X-ray diffractometer (XRD) from PANalytical B.V. was used from 8° to 30° 2 theta with a step size of 0.02. The samples were cleaned with distilled water and dried before testing. In the case of biodegradable polyesters, a few weeks of fluid exposure are necessary to reach the percolation threshold and notice pronounced effects in crystallinity. Crystallinity data from 4 to 16 weeks of fluid exposure was considered for analysis of in-vitro degradation effects.

#### Mechanical testing

Changes in mechanical integrity are a strong indicator of degradation in polymer blends. The changes in mechanical properties were studied by tensile tests, carried out on a Universal Testing Machine (Instron) with a 100 N load cell. Test specimens had a gauge length of 10 mm and width of 1.0 mm. Thickness values for each specimen were input prior to starting the test, in the BlueHill software. The test speed was set at 5 mm/ min. Five specimens were tested at each time point, and tensile strength (σ), strain at failure (ε) and Young’s modulus (E) were analyzed. Images of the fractured specimens were collected to understand the type of fracture. The type of fracture and clarity indicate changes in crystallinity of polymers, often supported by a change in the Young’s modulus.

### Statistical analysis

A Bartlett’s test was carried out to test homogeneity variances across each exposure-time point combination (example: PBS 4 weeks), for mechanical properties. Pairwise comparisons using Tukey’s procedure were carried out on rejecting the equality of means. P-value less than 0.05 suggested significant statistical differences.

### Consent for publication

Authors read and approved the final manuscript.

## Results

### Changes in crystallinity

**Figure 1:**
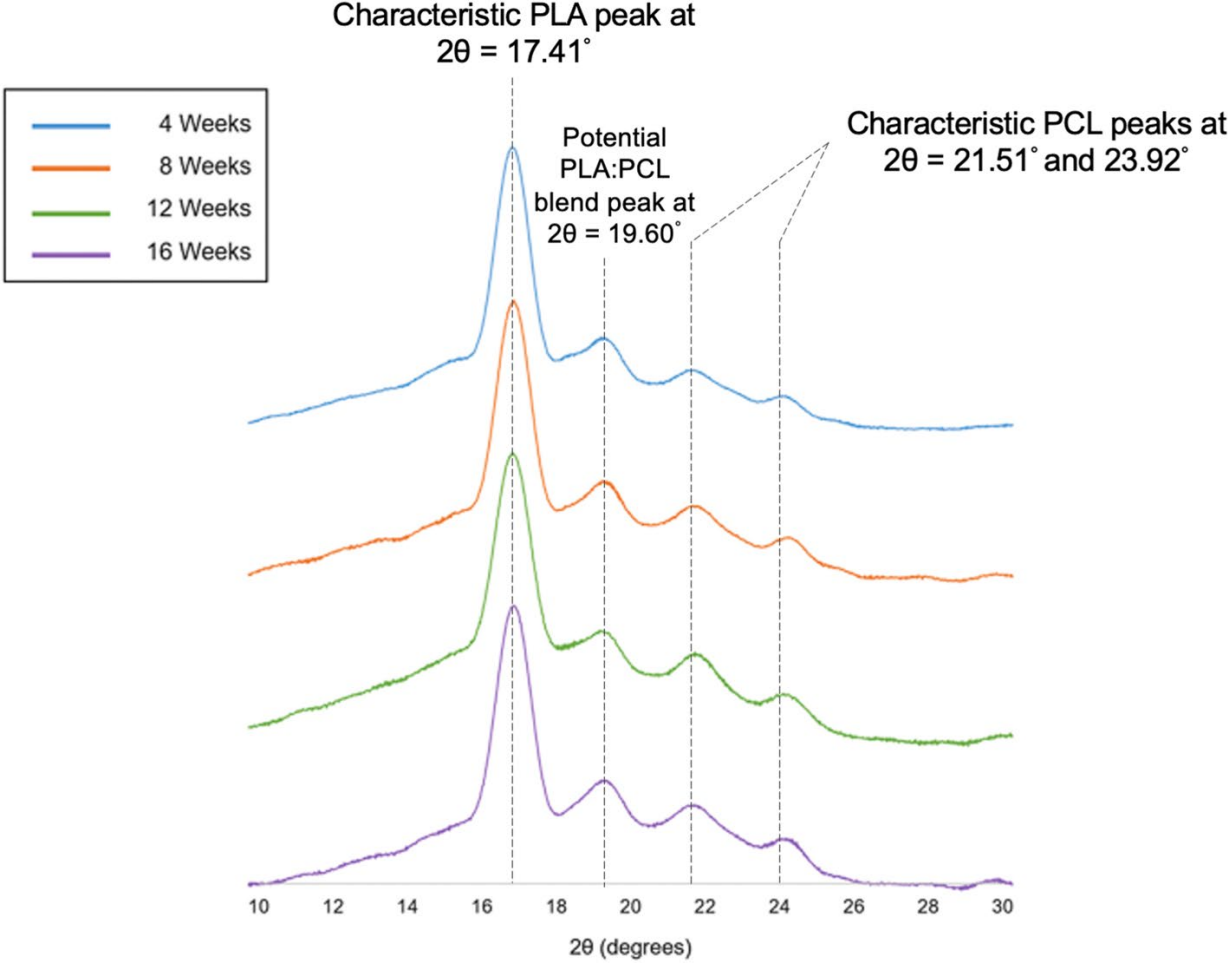
Crystallinity changes on exposure to phosphate buffered saline. Changes in crystallinity over time on exposure to phosphate-buffered saline for 4 weeks (Blue), 8 weeks (Orange), 12 weeks (Green) and 16 weeks (Purple).

**Figure 2:**
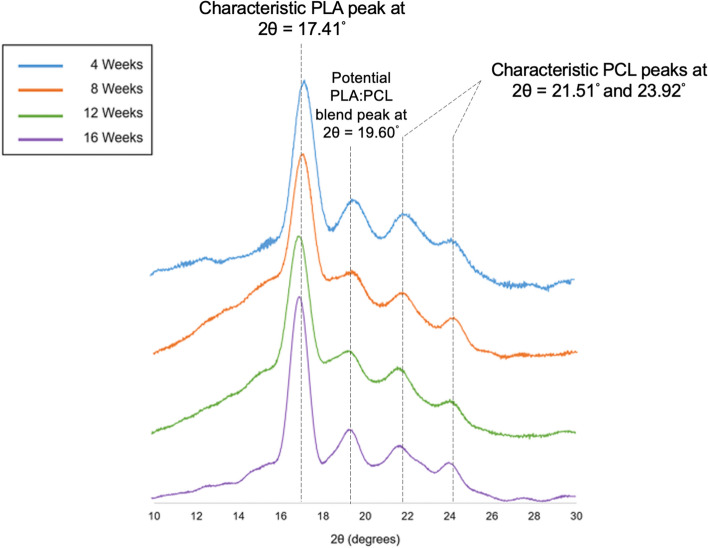
Crystallinity changes on exposure to amniotic fluid. Changes in crystallinity over time on exposure to amniotic fluid for 4 weeks (Blue), 8 weeks (Orange), 12 weeks (Green) and 16 weeks (Purple).

Crystallinity has a major influence on the rate of degradation in polymers. Depending on the type of polymer and exposure medium, the polymer or polymer blend can undergo degradation-induced crystallization and preferential rearrangements of crystalline domains. From the analysis of X-ray diffraction (XRD) data, peaks for PLA were observed at 2θ = 17.41°, and for PCL at 21.51° and 23.92° (Figs. 1 and 2), which are similar to values reported in literature^19^. Another peak was noticed at 19.60° (Figs. 1 and 2), which can be linked to the formation of semi-crystalline segments of PLA and PCL, entangled in the blend.

In XRD data from exposure to phosphate-buffered saline, no shift in peak positions was noticed over 16 weeks. An amorphous region was observed between 14° and 16° 2θ at the 4-week time point, which disappeared from 8 weeks of PBS exposure (Fig. 1).

On exposure to amniotic fluid, the peaks corresponding to PLA and PCL were visibly sharper at 16 weeks in comparison to the remaining time points. Also, at the 16-week time point, amorphous regions are noticed between 12° and 16°, and one of the PCL peaks displays an amorphous component between 22° and 23° (Fig. 2).

### Changes in mechanical properties

**Figure 3:**
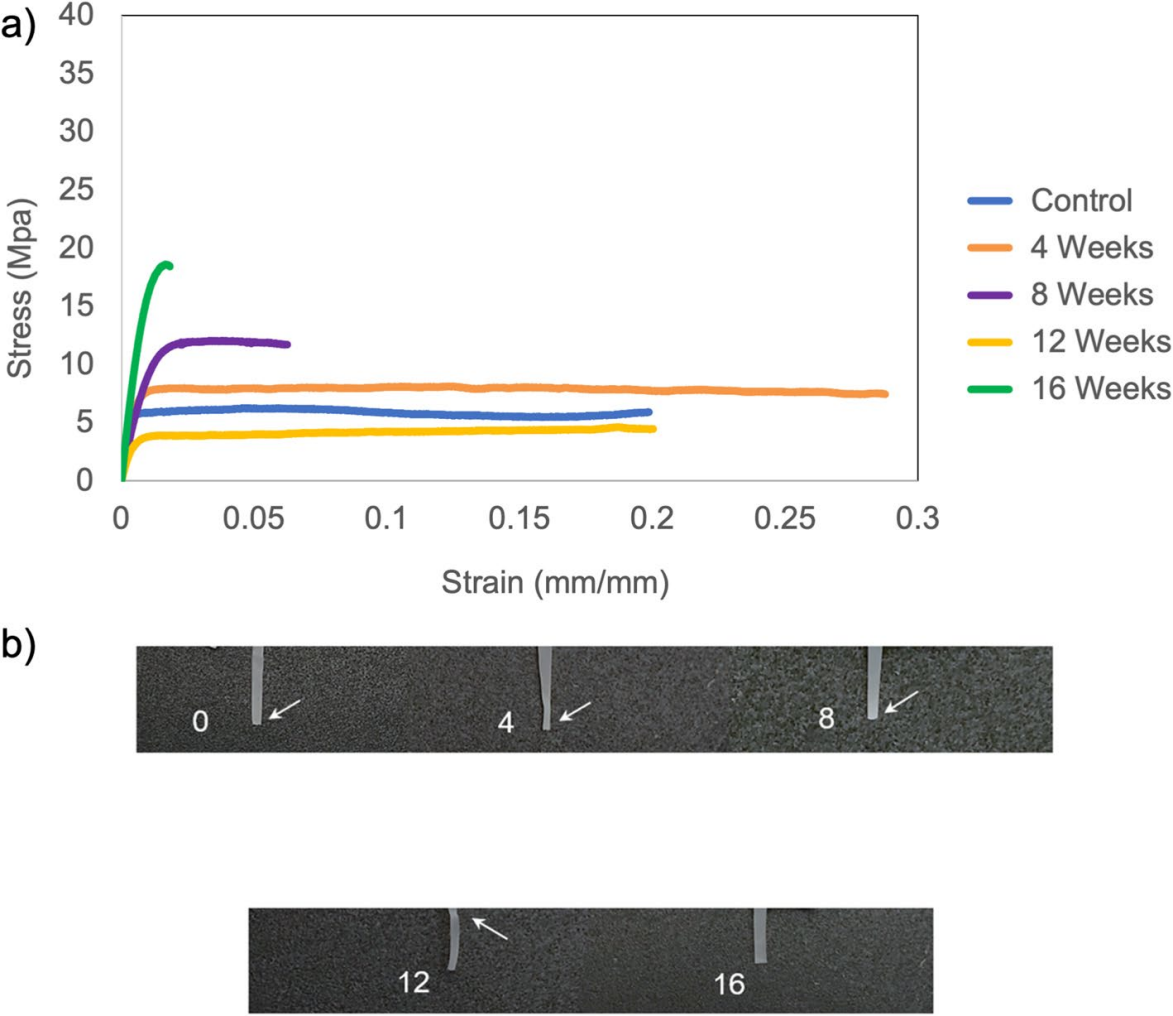
Mechanical integrity changes on exposure to phosphate buffered saline. (**a**) Representative stress–strain curves for control (0 weeks) and 4, 8, 12 and 16-week patch exposure to phosphate buffered saline. For each time point, the stress–strain curve of one specimen was chosen randomly out of the five specimens tested. These curves were plotted on the graph to study changes in the mechanical behavior of the patch, i.e. PLA-PCL blend. (**b**) White arrows indicate presence of necking phenomenon, i.e. stretching of the specimen at the point of fracture. Absence of white arrows indicates brittle fracture.

Studying changes in mechanical properties during in-vitro degradation enables us to foresee the patch performance upon in-vivo implantation for 16 weeks. The stress–strain curves representing each time point of phosphate-buffered saline exposure display a gradual ductile to brittle transition (Fig. 3a). Up to 12 weeks, the curves exhibit an initial elastic region followed by plastic-like behavior until the strain reaches the point of fracture. However, at the 16-week time point, the plastic region is completely absent, and the PLA-PCL blend exhibits brittle behavior. It is evident from the stress–strain curves that the elastic component in the blend at 8-week time point is greater than that of control and 4-week time point, which is a deviation from the observed trend (Fig. 3a). This can be attributed to inter-group variation, i.e. the diffusion of PBS took place at a faster rate in one out of the 5 specimens at 8-week time point, leading to greater crystalline domains and thus a higher modulus. Also, the average modulus at the 8-week time point (1375.39 ± 199.04 MPa) was lower than the average modulus for control (1848.98 ± 404.97 MPa) and the 4-week time point (1693.52 ± 146.6 MPa), establishing that the remaining 4 specimens at the 8-week time point had a lower modulus than those at control and 4-week time points.

Necking is characterized by a decrease in the specimen cross-sectional area at the point of fracture. This phenomenon is observed in polymers due to presence of disproportional localized strain. This is normally accompanied with strain hardening, leading to a white region near the fracture point. Strain hardening is caused by an increase in short-range order prior to fracture and is characteristic of ductile fractures in polymers. On exposure to PBS, necking and strain hardening was observed for all time points, except at 16 weeks (Fig. 3b). At 16 weeks, a relatively lower amount of whitening, i.e. strain hardening, was observed and no reduction in cross-sectional area was noticed at the point of fracture, i.e. necking (Fig. 3b). This signifies the onset of brittle behavior, as the polymer chains have attained the optimum configuration, and there is limited scope for rearrangements and chain mobility.

**Figure 4:**
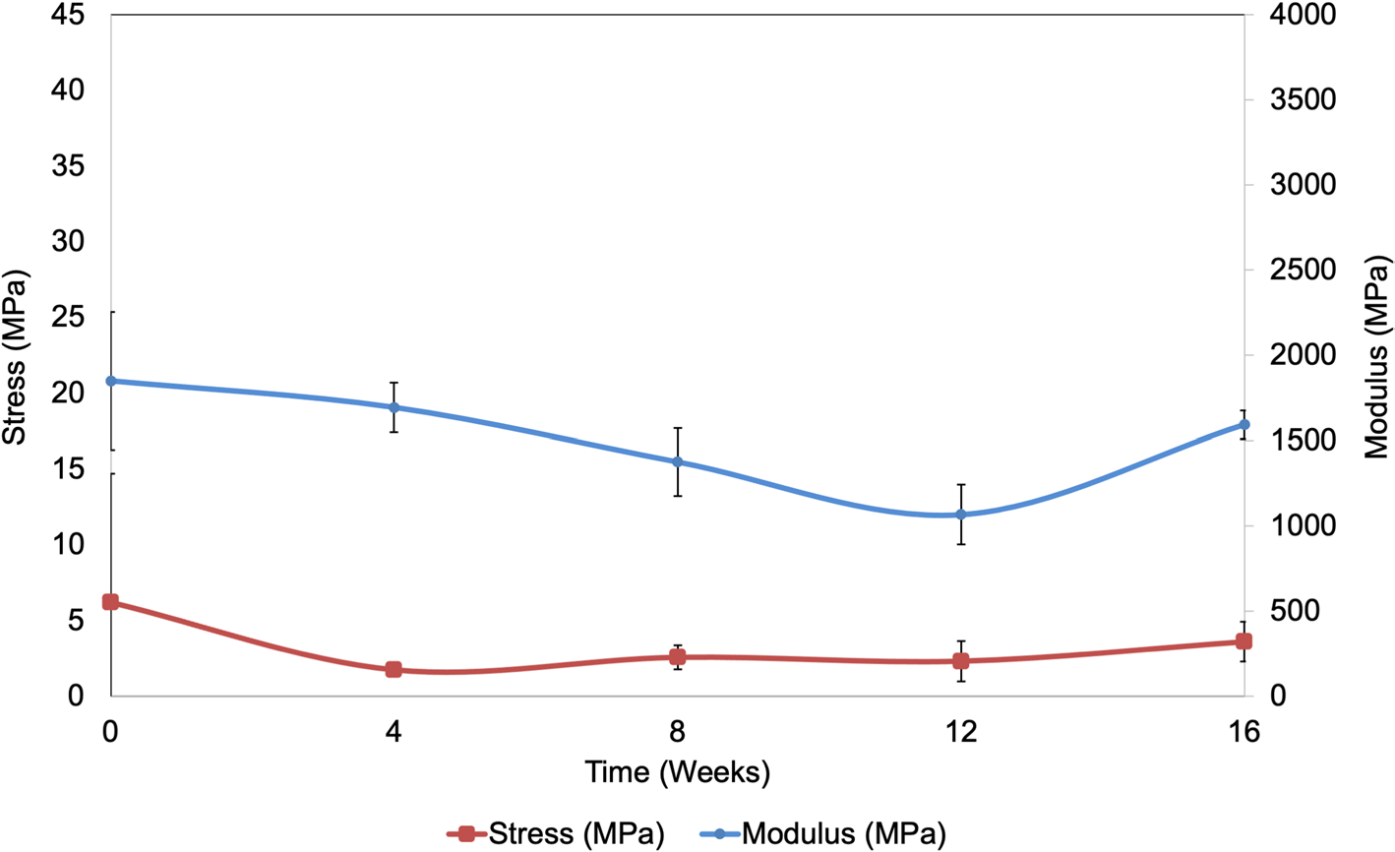
Average mechanical properties on patch exposure to phosphate buffered saline. Average values of tensile strength and Young’s modulus are displayed. Error bars report standard deviation (n = 5).

On analyzing the trend of mechanical properties over time for PBS exposure, the Young’s modulus decreases gradually and then increases at the 16-week time point (Fig. 4). For a confidence interval of 95%, statistical analysis indicates a significant difference in modulus (p = 0.024), with specific significance between control (0 weeks) and 12-week time point (p = 0.02) by pairwise comparisons. By 12 weeks, we observe ~ 63% strength loss, and an increase of strength by ~ 56% between 12 and 16 weeks. Compared to 12 weeks, the patch loses ~ 40% strain by 16 weeks, signifying the onset of brittle behavior. The end of plasticity and onset of brittle behavior is evident at 16 weeks from the stress–strain curves (Fig. 3a) and images of fractured tensile specimens (Fig. 3b).

**Figure 5:**
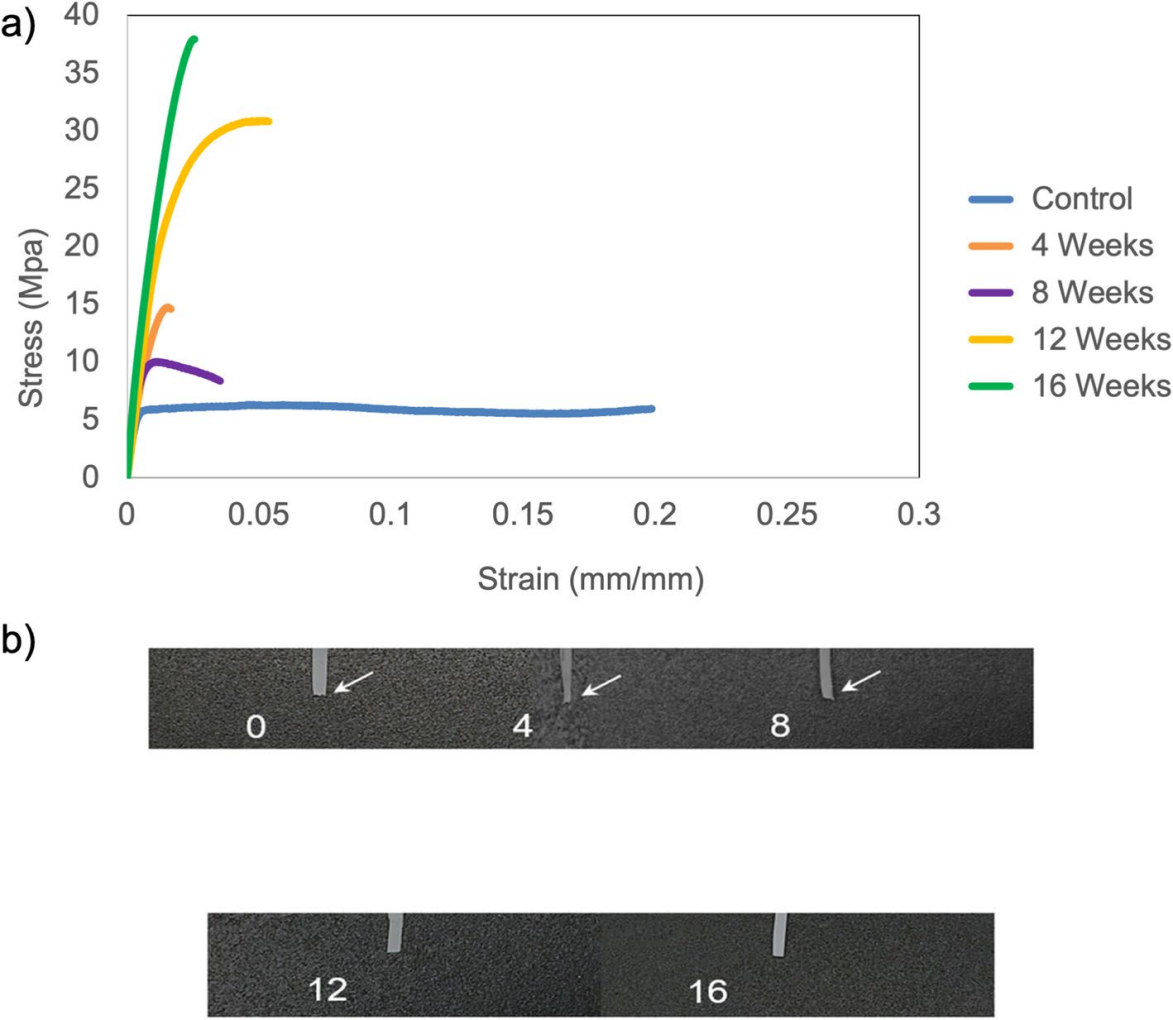
Mechanical integrity changes on exposure to amniotic fluid. (**a**) Representative stress–strain curves for control (0 weeks) and 4, 8, 12 and 16-week patch exposure to amniotic fluid. For each time point, the stress–strain curve of one specimen was chosen randomly out of the five specimens tested. These curves were plotted on the graph to study changes in the mechanical behavior of the patch, i.e. PLA-PCL blend. (**b**) White arrows indicate presence of necking phenomenon, i.e. stretching of the specimen at the point of fracture. Absence of white arrows indicates brittle fracture.

Exposure to amniotic fluid leads to an earlier onset of brittle behavior in the patch, i.e. PLA-PCL blend. Beyond the 8-week time point, stress–strain curves display a prominent increase in the elasticity, supported by a jump in the average stress for 12 and 16-week time points (Fig. 5). Though the stress–strain curves display a higher failure strain at the 12-week time point compared to the 4-week and 8-week time point, the elastic component is much greater for the 12-week time point. Greater amount of strain can be necessary to reorient the remaining disoriented fractions during transition to brittle behavior. At 16 weeks, the high elastic component and lower failure strain confirm a complete transition to brittle behavior. This observation is supported by X-ray diffraction data, which exhibits relatively narrow peaks at 16-week time point in comparison to peaks at 12-week time point (Fig. 2). Also, the average failure strain at the 12-week time point (6.73 ± 1.1%) was lower than the average failure strain at the 8-week time point (9.58 ± 4.66%), implying that the remaining 4 specimens at 12-week time point had a lower failure strain than those at the 8-week time point. The stress–strain curves at the 12-week and 16-week time points exhibit the absence of necking, which indicates a ductile to brittle transition (Fig. 5a).

The representative images of fractured specimens corroborate the type of fracture observed in stress–strain curves, at different time points of exposure to amniotic fluid. The absence of necking, i.e., localized change in cross-sectional area at the point of fracture, signifies brittle behavior of the patch (Fig. 5b). On 16 weeks of exposure to amniotic fluid, the specimen also turned opaque in comparison to specimens at previous time points (Fig. 5b). This establishes the high crystalline content in the patch, which will be discussed in detail in the next section.

**Figure 6:**
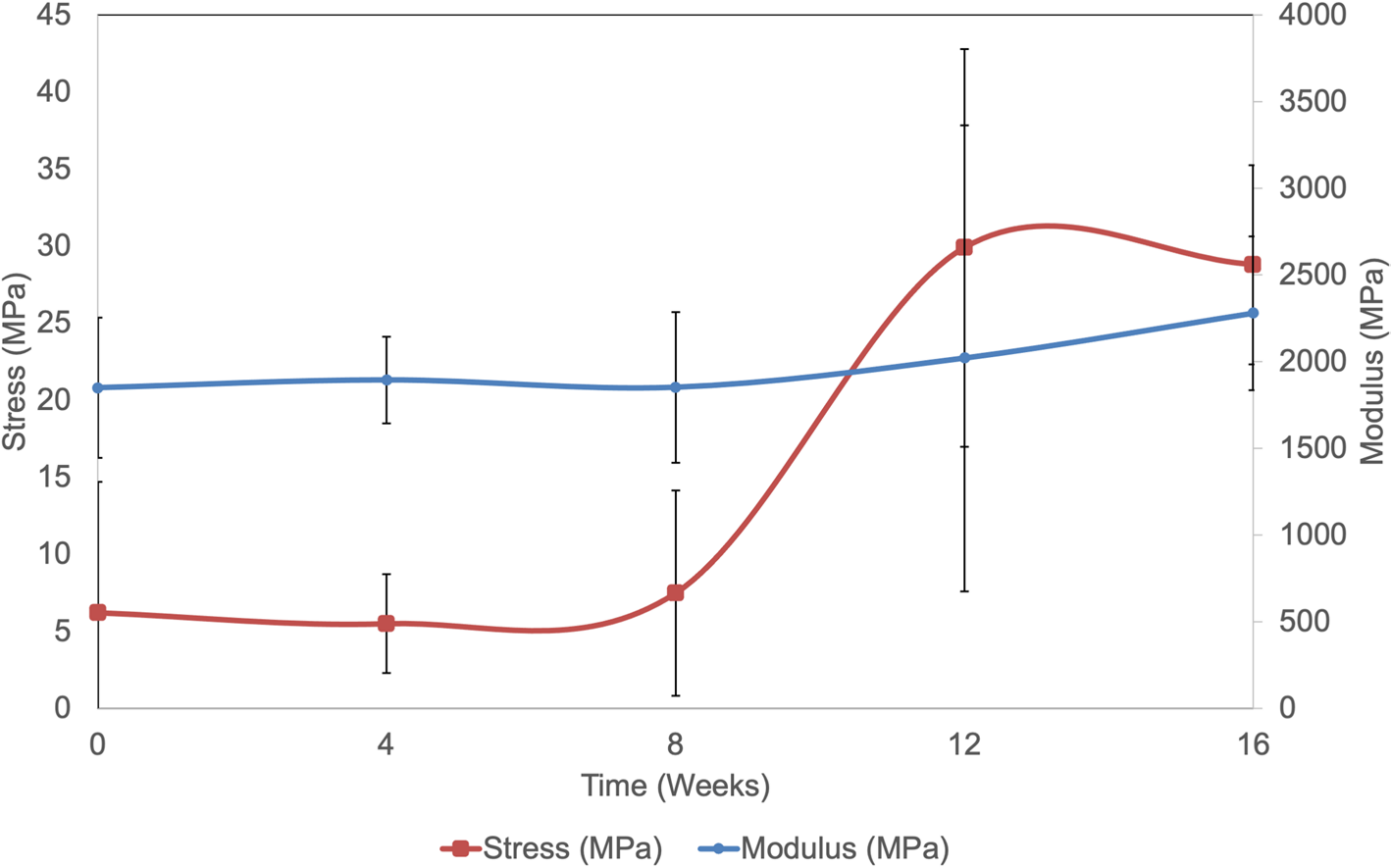
Average mechanical properties on patch exposure to amniotic fluid. Average values of tensile strength and Young’s modulus are displayed. Error bars report standard deviation (n = 5).

The moduli and strength display a gradual increase over time, on exposure to amniotic fluid (Fig. 6). By 16 weeks, there is ~ 300% increase in strength and ~ 74% decrease in strain. The large error bars at the 12-week time point are a result of one of the 5 specimens displaying excessively high strength and modulus (Fig. 6). However, this specimen was used in data analysis and calculations to maintain uniformity of data.

On visual investigation of the additional set of patch strips exposed to PBS and AF at 20 weeks, majority of the strips displayed central cracks and fractures along the cross-section. These strips were not subjected to any characterization, to avoid erroneous data and conclusions.

## Discussion

Poly(lactic-acid) (PLA) and poly(ε-caprolactone) (PCL) are also termed as ‘spine polymers’, due to their abundant use in spinal implants and related applications. Approved for hard- and soft-tissue repair by the U.S. Food and Drug Administration, PLA and PCL have been employed in several orthopedic implants and disc disorder treatments^20–24^. The slow-degrading PCL and fast-degrading PLA can be blended to attain excellent mechanical properties, shape memory characteristics and biocompatibility with reduced inflammatory response^25–28^. Our designed patch comprises of a PLA-PCL blend^14^, and the in-vitro degradation behavior will be explored in this study.

Biodegradable polymers employed in the designed patch enable gradual degradation after implantation, preventing costs and trauma associated with secondary patch removal procedures. The degradation scheme involves aqueous diffusion in the polymer matrix followed by hydrolytic degradation by water or enzymes. Ester group hydrolysis leads to chain scission and the formation of carboxylic and alcoholic end groups^18,22^. The designed patch is composed of a PLA-PCL blend, with the two polymers mixed in 83:17 weight ratios respectively^14^. A minor amount of PCL is known to accelerate the PLA hydrolysis in blend films, due to an increased concentration of terminal carboxyl groups^29^. To study in-vitro degradation, the designed patches were exposed to phosphate buffered saline (average pH 7.4) and amniotic fluid (average pH 8.26) separately. Amniotic fluid contains enzymes with varying activity levels based on the gestation period, however, there is no evidence of their participation in ester hydrolysis^30,31^. Our study did not attempt to investigate enzyme activity in the amniotic fluid used^32,33^.

Previous work conducted by our group documents the effect of in-vitro degradation on weight loss, surface roughness and functional groups^14^. It is critical to discuss these properties in this section as they corroborate the findings from crystallinity and mechanical property investigations and provide a holistic understanding of the in-vitro degradation profile. The trend of weight loss experienced by the designed patch was observed in earlier studies of PLA-PCL blends^14,29^. Basic media is known to accelerate the degradation, caused by more increased availability of base attack sites^18^. However, the difference in pH of PBS and AF did not significantly affect the weight loss at 16 weeks^14^. Thus, the presence of enzymes in AF failed to impact bulk degradation to the extent of impacting weight loss.

Modification of surface properties can influence the hydrolytic degradation of aliphatic polyesters^29^. Enzymes present in media modify the surface and physico-chemical characteristics^31^ but fail to impact the breakdown of PCL on implantation^34^. Surface erosion is a strong indicator of hydrolysis in PLA-based systems, corroborated by literature reporting a linear relationship between surface roughness and time on aqueous exposure^35,36^. From previous work conducted on the designed patches, amniotic fluid exposure caused greater changes in surface roughness compared to phosphate-buffered saline at 16 weeks^14^. Atomic force microscopy images taken at 16 weeks of AF exposure exhibited larger diameter surface craters, indicating the extent of surface impact and hydrolysis of susceptible PLA segments.

The chemical interactions in PLA-PCL blends are caused by hydrogen bonding between C=O group in PCL and terminal hydroxyl groups in PLA^37^. Polyester films immersed in basic aqueous solutions display absorbance peaks at 1570 cm^−1^, as reported in literature^38^. Similarly, exposure to phosphate-buffered saline causes an increase in area of peak at 1750 cm^−1^. This indicates greater number of carbonyl bonds, which are part of the terminal [COOH] end-group. A greater carboxylic acid end-group concentration results from chain scission during hydrolysis of PLA and PCL^39^. Accelerated hydrolysis takes place due to rising hydrophilicity, as more esters are converted to acid and alcohol end-groups^40^.

The participation of amniotic fluid enzymes in bulk ester hydrolysis has not been reported, however, the enzymes might impact the surface properties^31^. From previous work conducted on the designed patch, amniotic fluid exposure exhibited presence of carbonyl peaks and visible stretching of O–H peak at 16 weeks, compared to 16 weeks spectra on PBS exposure where minimal changes in the O–H peak were observed^14^. These findings indicate more terminal hydroxyl groups in the system, which can advance the rate of hydrolysis. Acceleration of relative hydrolytic surface degradation has been evidenced on exposure to basic media in other lower molecular weight lactide-based systems and might be similarly impacting the PLA domains in the designed patch^39^. Similar to the percolation phenomenon, the aqueous media reaches the bulk of the system only at 16 weeks, triggering the release of degradation products and creation of hydroxyl groups^40^.

Both PLA and PCL consist of crystalline and amorphous domains in varying content, based on molecular weight and crystallinity (Supplementary Fig. 2). However, it should be noted that PLA domains are major contributors to the crystalline content of the blend, owing to the low molecular weight and percentage of the PCL used in the blend. Studying the changes in crystallinity provides valuable information about internal rearrangements in the blend structure and can offer deep insight into the degradation behavior. Hydrolytic degradation is expedited by oligomeric products such as carboxylic acids within the blend matrix^41^. The increasing content of carboxylic acid-containing oligomers has been confirmed by results of FTIR-ATR tests from the previous study^14^.

On analyzing XRD data of PBS exposure, the disappearance of the amorphous region between 14° and 16° beyond the 4-week time point indicates bulk erosion and redistribution of domains. This occurrence at the 4-week time point can be linked to the initial penetration of aqueous media in the bulk of the system, followed by displacement of oligomeric products outwards from the core. Between the 4-week and 8-week time points, the exposure to 37 °C establishes chain mobility in PCL, triggering the recrystallization of these oligomeric products. This translates into relatively crystalline peaks observed at 8, 12 and 16-week time points.

XRD results on amniotic fluid exposure display a pronounced increase in crystalline domains at the 16-week time point. This can be noticed by the narrower peaks noticed at 16-week time point in comparison to peaks at the remaining time points, seen at 17.41°, 19.60°, 21.51° and 23.92° 2theta. The half-width of peaks relates to the crystallite dimensions, and the narrow half-width of peaks observed at 16-week exposure to amniotic fluid indicates the existence of large crystallites. The exposure to aqueous media at 37 °C leads to an annealing effect that contributes to the ordered arrangement of chains^42^. The increase in crystallinity of PCL domains can also be attributed to degradation-induced crystallization of amorphous domains in the matrix^43^. Pitt et al. reported a consistent but slower crystallinity increase beyond 4 weeks of PCL implantation in a rabbit model, which can be linked to crystallization of tie segments facilitated by chain cleavage in amorphous phase because of low glass transition temperature (− 60 °C) of PCL^42^. Similarly, the combined effect of temperature on PCL, and enzymes on PLA, accelerates the surface erosion of respective low molecular weight fractions, also supported by findings from surface roughness studies^14^. At the 16-week time point on XRD data, amorphous fractions are also visible between 12° and 16°, and an amorphous component is observed between 22° and 23°. This finding indicates the sequential separation of amorphous domains taking place alongside the formation of large crystallites in the blend matrix. This argument is supported by the ATR-FTIR data at 16 weeks of amniotic fluid exposure, which reports more terminal hydroxyl groups due to release of degradation products in the system^14^.

The change in mechanical integrity is a strong indicator of degradation in polymer blends. Brittle behavior at 16 weeks for PLA-PCL blends has been reported in previously published studies^41^. This phenomenon is also visible in the x-ray diffraction studies, where the crystalline peaks are retained at 16 weeks, even though the specimens have undergone considerable strength loss compared to the control (0 weeks) specimens. Additionally, the relative narrowing of peaks observed at the 16-week time point of amniotic fluid exposure consolidates the transition to brittle behavior.

Polymer chains react at a slow rate during hydrolysis, leading to chain cleavage and added mobility. This causes a gradual and prominent increase in crystalline domains, that eventually impacts mechanical integrity. Poly(lactic acid) exposure to a temperature of 37 °C creates an annealing effect that also impacts crystallinity. Due to this effect, the amorphous domains produce a spherulite microstructure consisting ordered lamellae inter-connected by short chains. Depending on the time of annealing, the spherulites occupy partial or complete volume of the polymer. This leads to two configurations of the amorphous domains: (a) fully grown spherulites and (b) partially grown spherulites with remaining volume occupied by ordered lamellae^44^. Tsuji et al. proposed that ordered lamellae inter-connected by short chains might have a higher density of terminal carboxyl and hydroxyl groups that do not participate in crystallization. This suggests that the two configurations of amorphous domains in PLA matrix can degrade at different rates, which might impact the crystallinity and mechanical properties^45^.

The low glass transition temperature of PCL (− 60 °C) could lead to significant chain mobility due to annealing. The chain mobility would rise further due to reduction of chain length during hydrolysis. This can lead to recrystallization and substantially contribute to the crystallinity of the polymer blend^46^. The impact of annealing on crystallinity of the blend can be directly linked to the mechanical behavior. When the blend displays ductile behavior, it indicates the presence of disoriented amorphous domains that can be further oriented into crystalline structures on the application of strain. However, the transition of ductile to brittle behavior signifies negligible disorientation in the blend, and an optimal content of crystalline domains.

As the designed patch is unique in terms of the composition and functional properties, it is less likely to conduct an adequate comparison to commercialized products individually, as the new patch collectively possesses the claimed features that are superior to one or certain, if not all, unmet needs of the conventional patches. For example, cellulose-based patches are known to be porous, indicating the permeability will be different compared to the designed patch and thus comparative mechanical testing will not lead to a uniform comparison. However, mechanical properties of the designed patch were compared to values reported in literature to understand the range of mechanical response. The designed patch has modulus values that are much higher than commercially used patches, while the tensile strength is higher, and in some cases lower than the commercially used patches^47^. However, the key metric for the suitability of an artificial fetal MMC patch is the similarity to mechanical properties of cranial human dura matter. The tensile strength of the designed patches was similar to that of cranial human dura matter^47^ and remained stable during 16 weeks of exposure in PBS and amniotic fluid.

Due to unavailability of published data on polymer degradation in amniotic fluid, phosphate-buffered saline was used as a reference, and also to emulate body fluids. Degradation of different polymer systems in PBS has been studied before, but the degradation kinetics of our PLA-PCL blend needed to be investigated when subjected to simulated fetal conditions of movements and temperature. The continuous rotations and vibrations will cause rigorous movement of fluid in the tubes, which would strongly interact with the immersed patch strips. Due to these interactions, the fluid might generate a cyclic stress on the patch, that can influence the rate of hydrolysis.

On review of mechanical testing results, exposure to both media, i.e. phosphate-buffered saline and amniotic fluid, led to a brittle fracture of the PLA-PCL blend at 16 weeks. Though the mechanical integrity of the patch does not deteriorate at 16 weeks, all the above characterizations indicate the progress of hydrolytic degradation. Lastly, crack initiation and physical disintegration of additional sets of patches observed at 20 weeks of PBS and AF exposure consolidate the in-vitro biodegradability of the designed patch. This can be an effect of the simulated fetal environment and the interaction with the amniotic fluid cells, causing accelerated disintegration of the patch. Our previous experiments in the PLA/PCL patch showed biocompatibility as a cell substrate in vitro that could help during the healing processes in vivo^14^ but also affect the degradation of the designated patch. Further studies are needed to evaluate the degradation effect and cell interaction.

The onset of brittle behavior from 12 weeks of amniotic fluid exposure and 16 weeks of phosphate-buffered saline is evident from the mechanical testing data. This does not comply well with the flexibility required in the patch to incorporate the radial expansion during fetal growth. However, an accurate mechanical response of the designed patch can be gauged via in-vivo implantation in animal models, where the patch would simultaneously experience contact with physiological fluid and amniotic fluid, in addition to temperature and fetal movements. Modifications in the blending technique and addition of branching agents to improve the flexibility of the patch will be explored, which will help enhance the comfort of the surgeon and compliance of the patch to adapt to the spinal canal during prenatal repair of spina bifida.

## Conclusions

This study focusses on crystallinity and mechanical property changes of the designed patch for intended use in prenatal open or fetoscopic myelomeningocele repair, when exposed to a simulated fetal or amniotic environment. Crystallinity studies indicated the presence of oligomeric products at 16 weeks of amniotic fluid exposure and the existence of large crystallites in the blend matrix. These observations were supported by functional group analyses from our previous studies^14^. The mechanical testing data establishes that patch integrity does not deteriorate during 16 weeks exposure to amniotic fluid. The range of characterizations indicate the progress of hydrolytic degradation in the patch. Future work will consist of long-term implantation of the designed patch in spina bifida large animal models for assessment of the in-vivo degradation profile and analysis of the adaptability of the patch and the ergonomics of the surgery.

## Supplementary Information

Supplementary Legends.

Supplementary Figure 1.

Supplementary Figure 2.

## Data Availability

Data supporting the findings of this study are available within the article and its supplementary information.

## Abbreviations

PBS: Phosphate-buffered saline
AF: Amniotic fluid
PLA: Poly(lactic acid)
PCL: Poly(ε-caprolactone)
XRD: X-ray diffraction
FTIR-ATR: Fourier transform infrared spectroscopy-attenuated total reflectance
