# Supplementary material for: Biodegradation of poly(l-lactic acid) and poly(ε-caprolactone) patches by human amniotic fluid in an in-vitro simulated fetal environment
Source: Sci Rep. 2022 Mar 10;12:3950. doi: 10.1038/s41598-022-07681-8 (PMC8913814; doi:10.1038/s41598-022-07681-8)
Supplement: Supplementary file 1 — Supplementary Legends. [file 41598_2022_7681_MOESM1_ESM.docx]

**Biodegradation of Poly (l-lactic acid) and Poly (ε-caprolactone) Patches by Human Amniotic Fluid in an In-vitro Simulated Fetal Environment**

Rigwed R. Tatu^1^, Marc Oria^2,3,4^, Marepalli B. Rao^5^, Jose L. Peiro^2,3,4^, Chia-Ying Lin^1,6,7^

**Supplementary Figure Legend:**

**Supplementary Fig. 1: Experimental Setup**

Patch strips submerged in Phosphate Buffered Saline and Amniotic Fluid and placed on a shaker inside an oven at 37^o^C for 16 weeks. Both fluids were changed and the material properties of the patch strips were analyzed at 4, 8, 12 and 16 weeks.

**Supplementary Fig. 2:** Schematic representation of PLA and PCL domains in the patch, i.e. PLA-PCL blend
