# Supplementary figures and images for: Biodegradation of poly(l-lactic acid) and poly(ε-caprolactone) patches by human amniotic fluid in an in-vitro simulated fetal environment
Source: Sci Rep. 2022 Mar 10;12:3950. doi: 10.1038/s41598-022-07681-8 (PMC8913814; doi:10.1038/s41598-022-07681-8)

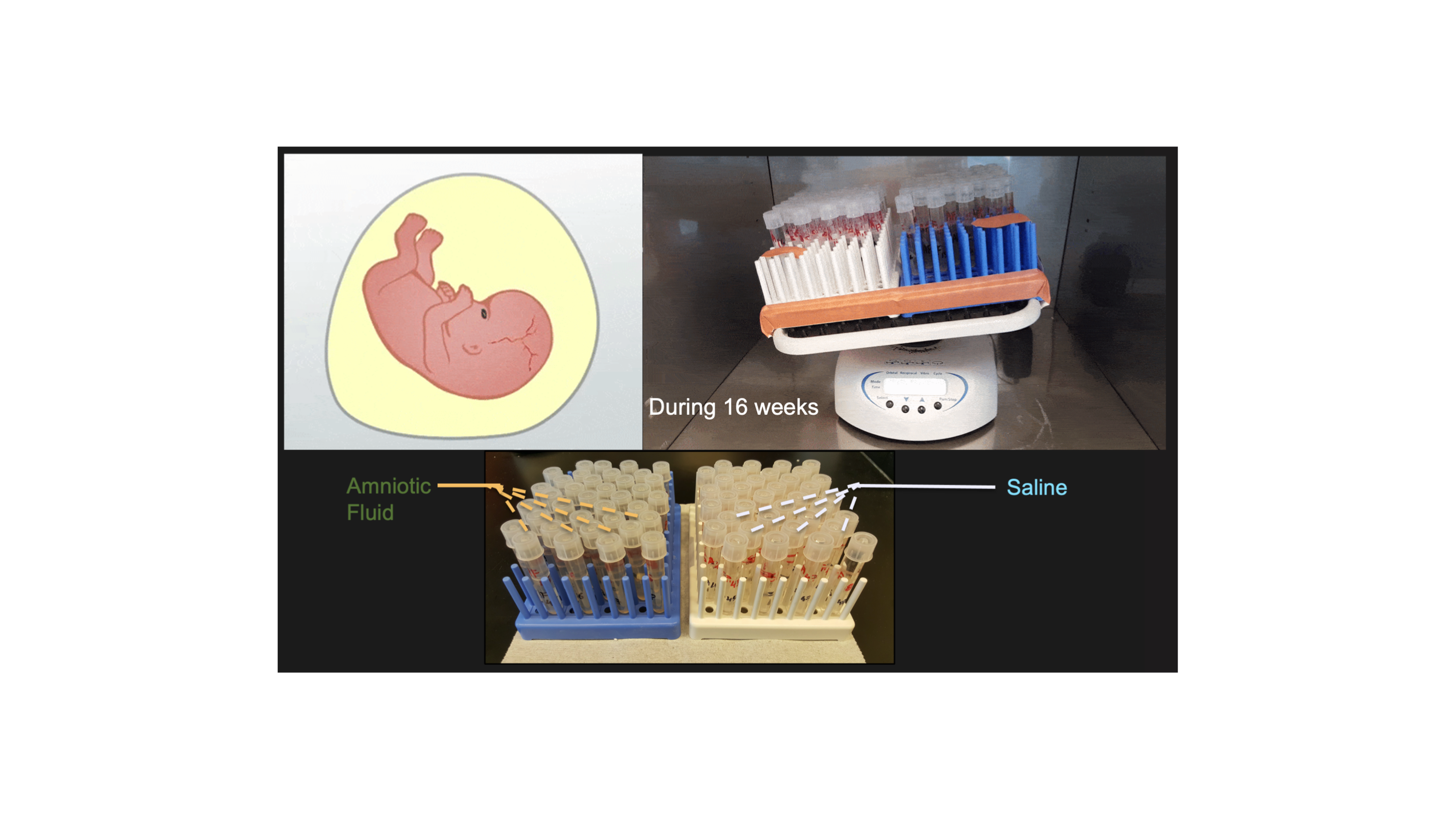

Supplement: Supplementary file 2 — Supplementary Figure 1. [file 41598_2022_7681_MOESM2_ESM.tiff]

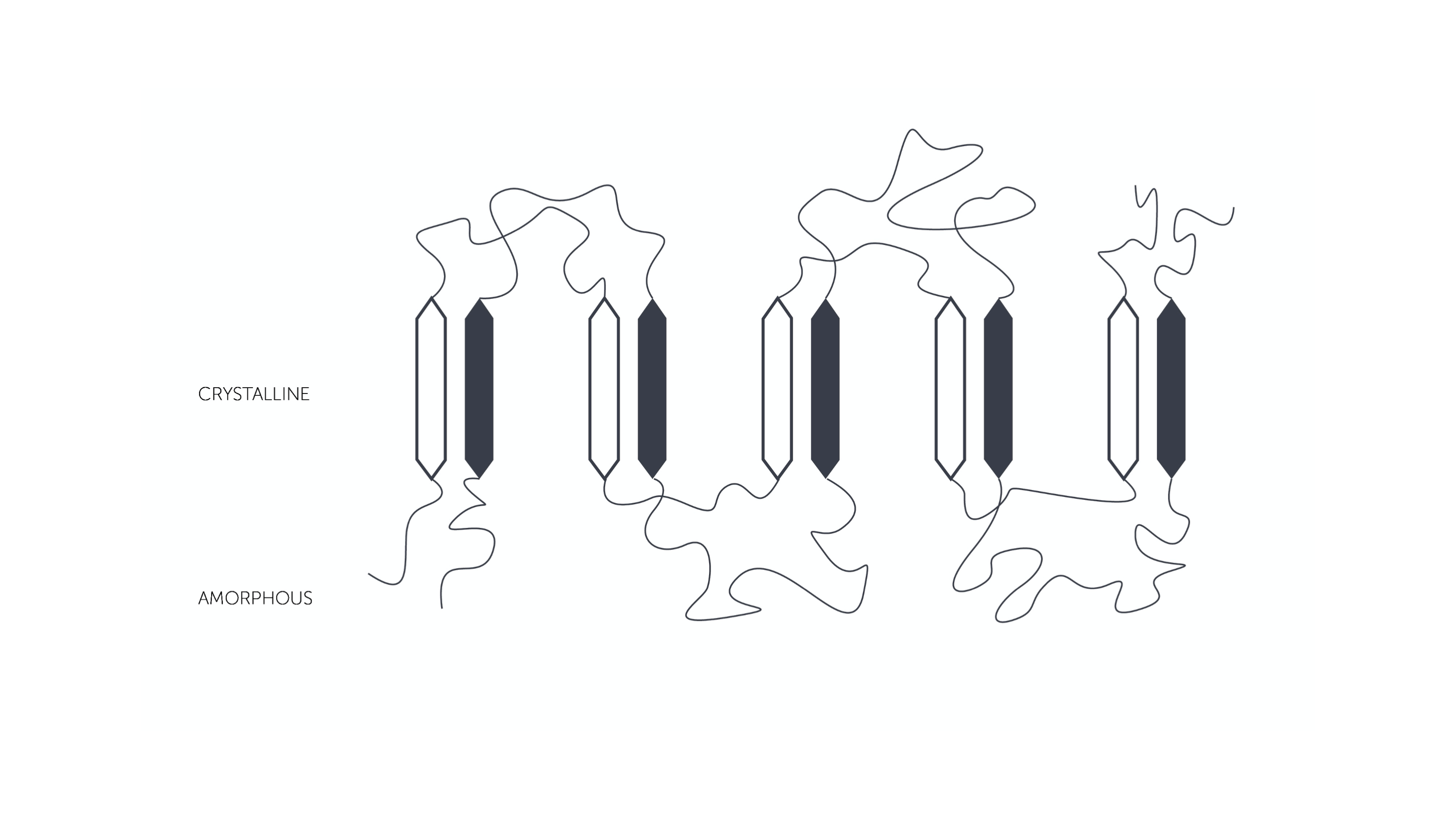

Supplement: Supplementary file 3 — Supplementary Figure 2. [file 41598_2022_7681_MOESM3_ESM.tiff]
